# Medication reconciliation by pharmacists for pre-admission patients improves patient safety

**DOI:** 10.1186/s40780-024-00340-2

**Published:** 2024-04-26

**Authors:** Yunami Yamada, Ryo Kobayashi, Taishi Yamamoto, Hironori Fujii, Hirotoshi Iihara, Kato-Hayashi Hiroko, Shohei Nishida, Ryo Hoshino, Takashi Niwa, Keisuke Kumada, Masahito Shimizu, Akio Suzuki

**Affiliations:** 1 Department of Pharmacy, Gifu University Hospital, Gifu, Japan; 2 Laboratory of Advanced Medical Pharmacy, Gifu Pharmaceutical University, Gifu, Japan; 3 Patient Safety Division, Gifu University Hospital, Gifu, Japan; 4 Department of Emergency & Disaster Medicine, Gifu University Graduate School of Medicine, Gifu, Japan; 5 Department of Gastroenterology, Gifu University Graduate School of Medicine, Gifu, Japan

**Keywords:** Medication reconciliation, Patient safety, Medication error, Pre-admission

## Abstract

**Background:**

Medication errors related to the pre-admission medication history obtained on admission are a major cause of medication error during hospitalization. Medication reconciliation (MR) improves patient safety through the detection of inadvertent medication discrepancies at transitions of care. The aim of this study was to evaluate the effect of MR by pharmacists for patients prior to hospital admission on the incidence of medication errors in the early post-admission period.

**Patients and methods:**

Patients admitted to the orthopedic ward for surgery between April 2012 and March 2020 were included. Pharmacist-led MR for pre-admission patients was started on April 1, 2017. The incidence of medication errors related to pre-admission medications that occurred during hospitalization were compared between the pre- and post-initiation of pharmacist-led MR (pre-initiation: April 1, 2012 to March 31, 2015, post-initiation: April 1, 2017 to March 31, 2020).

**Result:**

In the post-initiation group, 94.2% (1245/1321) of patients who were taking medications on admission had a pharmacist-led MR before admission. The proportion of patients whose physicians ordered the prescription of their pre-admission medications at the time before hospitalization to continue from admission was significantly higher in the post-initiation group than in the pre-initiation group (47.4% vs. 1.0%, *p* < 0.001). The incidence of medication errors related to pre-admission medications during hospitalization was significantly lower in the post-initiation group than in the pre-initiation group (1.83% vs. 0.85%, *p* = 0.025). Pharmacist-led MR prior to admission was a significant protective factor against incidents related to pre-admission medication (odds ratio (OR), 0.3810; 95% confidence interval (CI); 0.156–0.9320, *p* = 0.035).

**Conclusion:**

Pharmacist-led MR for patients prior to hospital admission led to a reduction in medication errors related to pre-admission medications during hospitalization. Patient safety during hospitalization can be improved by accurate medication histories provided early by pharmacists.

## Introduction

Medication reconciliation (MR) is defined by the World Health Organization as “the formal process in which health care professionals partner with patients to ensure accurate and complete medication information transfer at interfaces of care” [1]. On admission to hospital, healthcare professionals should obtain a Best Possible Medication History (BPMH) of all medications taken prior to admission and use this for inpatient prescriptions. Several international patient safety organizations, such as the World Health Organization (WHO) [1], the Joint Commission International (JCI) [2] and the Institute for Health Care Improvement (IHI) [3] have recognized MR as an important process for improving patient safety by identifying unintentional medication discrepancies (UMD) at transitions of care. A BPMH is the cornerstone of the medication reconciliation process. The collection of complete medication lists prior to admission is expected to contribute to the prevention of medication errors.

Nevertheless, medication history errors, namely omission or commission errors at the time of hospital admission, remain common, and occur in up to 67% of cases [4]. One study found that in the 85% of patients who experienced errors in medication prescribing at the time of admission, the cause was attributable to errors in their drug history [5]. An exploratory case study of 30 patients reported that the majority of medication errors occurred on admission to hospital, and that half of these errors were attributed to incomplete medication lists on the admission form [6]. Other studies have found discrepancies in medication histories between those completed by physicians on admission and those completed by pharmacists according to a systematic approach [7, 8]. These findings indicate that pharmacy-led MR is associated with a reduction in patient harms, such as medication discrepancies and medication errors [9]. It has also been reported that pharmacy-led MR at hospital admission reduces the length of hospitalization and in-hospital mortality [10].

Of note, these various reports investigated the impact of post-admission pharmacist-led MR. To our knowledge, however, the impact of pre-admission pharmacist-led MR on patient safety during hospitalization has not been reported. In 2017, our hospital established the Total Patient Support Center to obtain patient information and provide guidance to patients prior to admission. This center initiated the practice of pre-admission MR that year with the aim of facilitating admission and reducing workload after admission. Activities include obtaining patient information, in which pharmacists obtain medication history from patients and intervene in their medication, such as washing out antithrombotic drugs before surgical treatment.

The aim of this retrospective study was to assess the practice of MR by pharmacists for patients prior to admission and to determine its impact on the occurrence of medication errors related to pre-admission medications during hospitalization.

## Methods

### Patients

This study was conducted under a single-center, retrospective observational design at the 614-bed Gifu University Hospital. The study was conducted in the orthopedic ward for the following reasons: 1. patients admitted to the orthopedic ward are relatively elderly and often take medications for comorbidities in addition to orthopedic medications; 2. patients rarely change their pre-admission medications for comorbidities during hospitalization; 3. patients are admitted for relatively long stays for surgery and rehabilitation and require repeat prescriptions for medications which they were taking prior to hospitalization. The study population consisted of patients admitted to the orthopedic ward from April 1, 2012 to March 31, 2015 (pre-initiation of pharmacist-led MR) and from April 1, 2017 to March 31, 2020 (post-initiation of pharmacist-led MR). MR conducted by pharmacists for pre-admission patients was started from April 1, 2017 at the Total Patient Support Center, which is an outpatient unit in our hospital. Patients who were hospitalized multiple times during the study period, both before and after the introduction of pharmacist-initiated MR, were analyzed as a single-admission patients. The period from April 2015 to March 2017 covered the preliminary operation period of this center and was therefore excluded from analysis. In this period, pharmacist-initiated MR was implemented only for a portion of preadmission patients, as part of the study period established in preparation for the opening of the center. In addition, several changes were made to the pharmacist medication history reporting format during this period. Accordingly, uniform assessment of the effectiveness of MR during this period is difficult. Data were obtained from electronic patient medical records held in the central database of our hospital and retrospectively analyzed.

This study was carried out in accordance with the Ethics Committee of Gifu University Graduate School of Medicine and Gifu Pharmaceutical University and was approved by the Gifu University Graduate School of Medicine Review Committee (Institutional Review Approval Number 2023–103) and Gifu Pharmaceutical University (Institutional Review Approval Number 5–17). All procedures performed in studies involving human participants were in accordance with the ethical standards of the institutional and/or national research committee and with the 1964 Helsinki Declaration and its later amendments or comparable ethical standards. We posted information about the trial and how patients could opt out on the hospital’s website.

### Intervention of pharmacists for pre-admission patients

**Fig. 1:**
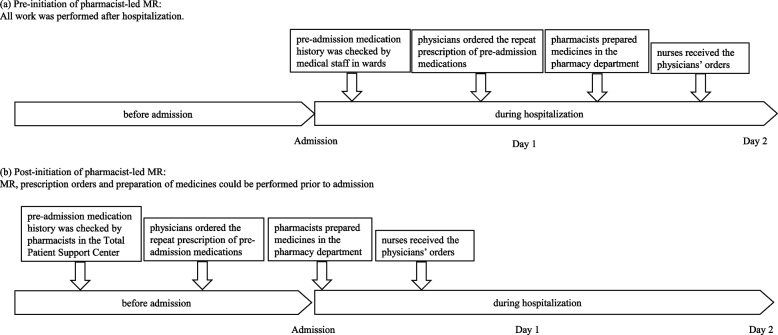
Schema of (**a**) pre- and (**b**) post-initiation of pharmacist-led MR, showing ordering of prescriptions, preparation of medicines in the pharmacy, and receipt of physician orders

Before initiation of pharmacist-led MR, the pre-admission medication history of inpatients was checked by medical staff, including physicians, nurses and pharmacists, after admission on Day 1, and physicians ordered the prescription of pre-admission medications which were to continue during hospitalization (Fig. 1A).

After initiation of pharmacist-led MR, all patients scheduled to be admitted, except those with emergency hospitalization or transfer from another hospital, received admission guidance in the Total Patient Support Center a few days to weeks before hospitalization (Fig. 1B). Pharmacists in this center obtained information on the medication history of the patients. If the patient did not bring a source of information (e.g. a list of medicines), it was collected by the pharmacist from the patient’s family doctor or pharmacy, with the patient’s consent. Pre-admission medication information was stored in the patient's electronic medical record as a provisional prescription order and could be used secondarily by the physician to order medication for the patient during hospitalization. In addition, pharmacists provided information on alternative medicines to physicians. Based on this information, physicians were able to understand the difference between the pre-admission medication and those medicines employed in the hospital. When there was a change in medication, the pharmacist changed the entry to reflect the latest medication history in the ward after admission.

For patients on antithrombotic therapy, pharmacists in the Total Patient Support Center ensured that appropriate withdrawal periods were calculated and, if necessary, ordered the withdrawal of antithrombotic medication under the direction of physicians.

### Comparison between pre- and post-initiation of interventions by pharmacists

Among patients who were taking medications before admission, the proportion of patients who continued their pre-admission medication during hospitalization, and the proportion of these patients for whom physicians ordered the continuation of prescription of their pre-admission medications before hospitalization were compared between the pre- and post-initiation of pharmacist-led MR at the Total Patient Support Centre. Assessing the impact of pharmacist-led MR on pre-admission medications requires the evaluation of incidents related to pre-admission medications that occur early after admission. This is because pre-admission medications need to be continued, and instructions for continuing prescriptions, and repeat prescriptions for pre-admission medications, are written early after admission. Therefore, the primary study outcome was the incidence of medication errors which were related to pre-admission medication and which occurred within 5 days after admission to the hospital. The incidence of medication errors was compared between the two groups.

### Preoperative interruption of antithrombotic medicine

The number of antithrombotic interruptions suggested to physicians and the acceptance proportion of these suggestions were compared for patients whose antithrombotic medication was interrupted preoperatively before and after initiation of the pharmacist-led MR in the Total Patient Support Center.

### Statistical analyses

Statistical analyses were conducted using R software version 3.5.1 (www.r-project.org). *P*-values less than 0.05 were considered significant. The primary study outcome was a comparison of the pre- and post-initiation of pharmacist-led MR using the chi-squared test. Multivariate logistic regression analysis was performed to estimate factors associated with incidents related to preadmission medications. To avoid overfitting based on the number of incidents, three factors were used: “Pharmacist-led MR prior to admission”, “prescriptions for medications taken prior to admission”, and “patient age”. “Prescriptions taken before hospitalization” was used as an indicator of early prescribing. “Patient age” was used as a surrogate for the number of medications taken. The number of drugs taken increases in patients aged ≥ 75 years [11].

## Results

### Patient demographics

Patient demographics pre- and post-initiation of pharmacist-led MR are shown in Table 1. The number of inpatients in the post-initiation group was higher than in the pre-initiation group (pre-initiation, 1041 patients; post-initiation, 1649 patients). Male/female ratio and median age were similar between the two groups. The proportion of patients who were taking medications before admission was 76.8% (800/1041) and 80.1% (1321/1649) before and after initiation of the pharmacist-led MR, respectively.

**Table 1:** Patient demographics in pre- and post-initiation of pharmacist-led MR for preadmission patients

	Pre-initiation of intervention	Post-initiation of intervention
Number of inpatients, n	1041	1649
Gender (male/female)	443/598	732/917
Age, median, years (minimax)	62 (4–95)	65 (6–99)
Number of patients receiving pre-admission medications, n (%)		
Presence	800 (76.8)	1321 (80.1)
Absence	241 (23.2)	328 (19.9)

### Interventions of pharmacists for pre-admission patients

The provision of MR by pharmacists for pre-admission patients started on April 1, 2017. Of patients who were taking medications before admission, 94.2% (1245/1321) in the post-initiation group received MR by a pharmacist prior to admission (Table 2). Among these patients, information about in-hospital alternative medications was provided by pharmacists to other medical staff, including physicians and nurses, via the electronic medical record for all except four patients (93.9%; 1241/1321). The median time from pharmacist-led MR to hospital admission was 13 days (25-75th percentile: 7–21 days). Most patients were admitted to the hospital within about 2 weeks of confirmation, suggesting that drug changes were infrequent. After initiation of the pharmacist-led MR, 306 patients who were taking antithrombotic medicine before admission were required to interrupt this antithrombotic medicine before admission because of pending surgical treatment (Table 3). In 69.9% (214/306) of these patients, the pharmacist confirmed that the physician’s previous withdrawal instructions were appropriate. In the remaining 30.1% (92/306), the pharmacist recommended to the physician that they interrupt the antithrombotic medication. This recommendation was accepted in 95.7% (88/92) of cases. Interruption of antithrombotic medicine in preoperative patients is shown in Table 4.

**Table 2:** Interventions by pharmacists for pre-admission patients (Medication reconciliation)

Number of inpatients, n	1649	
Number of patients receiving pre-admission medications, n	1321	
Medication reconciliation, n (%)		
Yes	1245	(94.2)
No	76	(5.8)
Provided information on alternative drugs, n (%)		
Yes	1241	(93.9)
No	80	(6.1)
Patient instruction, n (%)		
Yes	728	(55.1)
No	593	(44.9)

Each percentage refers to the number of patients receiving pre-admission medications

**Table 3:** Interventions by pharmacists for pre-admission patients (Interruption of antithrombotic drugs before surgical treatment)

Number of patients required to interrupt antithrombotic medicine before surgical treatment, n	306	
Pharmacist suggestions, n (%)	92	(30.1^a^)
Accepted suggestions, n (%)	88	(95.7^b^)

^a^Percentage of the number of patients required to interrupt antithrombotic medicine before surgical treatment

^b^Percentage of pharmacist suggestions

**Table 4:** Interrupted antithrombotic medicines in preoperative patients

	Pre-initiation of intervention	Post-initiation of intervention
Number of patients required to interrupt antithrombotic medicine before surgical treatment	139	306
Limaprost alfadex	50	86
Aspirin	47	86
Clopidogrel	18	35
Prasugrel	0	2
Cilostazol	10	17
Sarpogrelate	8	4
Ticlopidine	4	4
Dipyridamole	1	2
Warfarin	23	24
Rivaroxaban	3	19
Apixaban	0	17
Edoxaban	0	12
Dabigatran	4	3
Beraprost	6	8
Dilazep	0	9
Eicosapentaenoic acid	7	29

Includes patients taking more than one of the above drugs as a regular medication

### Proportions of physicians’ prescription orders entered during the hospital stay among patients who had received pre-admission medications

**Fig. 2:**
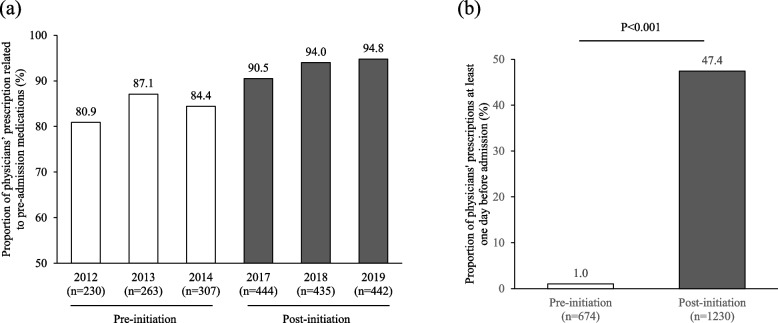
**a** Proportion of physician prescriptions related to pre-admission medications during hospitalization. **b** Proportion of physician prescriptions at least one day before admission for patients who had received pre-admission medication

As shown in Fig. 2a, the proportion of patients who were taking medicines before admission and who continued to take any medicine during hospitalization was at least 80% in both periods. Of these patients, the proportion of patients for whom physicians ordered the continued prescription of medications used before hospitalization was significantly higher in the post-initiation group than in the pre-initiation group (47.4% vs 1.0%, *p* < 0.001, Fig. 2b). In cases in which a patient did not receive a physician prescription during hospitalization for a medication that was entered pre-admission, the following reasons were identified: determination that continuation of the medication was no longer necessary; medication was changed; or instruction to continue taking a medication that had been prescribed by the family doctor prior to hospitalization.

### Incidence of medication errors which were related to pre-admission medication and occurred in the early phase of hospitalization

**Fig. 3:**
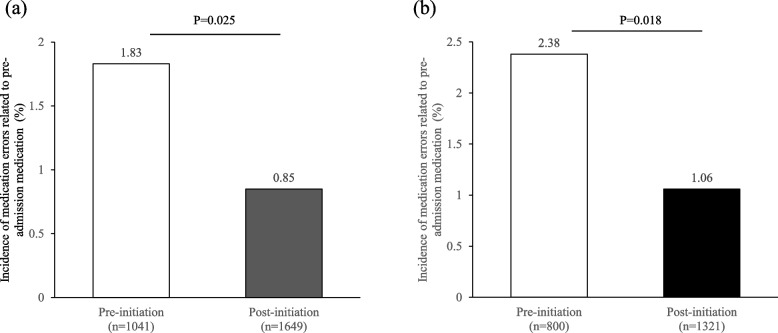
Incidence of medication errors related to pre-admission medication and which occurred within 5 days after admission (**a**) in all hospitalized patients, and (**b**) in patients who received pre-admission medication only

In all hospitalized patients, the incidence of medication errors related to pre-admission medication was significantly lower in the post-initiation group than in the pre-initiation group (1.83% *vs*. 0.85%, *p* = 0.025, Fig. 3a). Further, after excluding patients without pre-admission medication, the incidence of medication errors was lower in the post-initiation group than in pre-initiation group (2.38% vs. 1.06%, *p* = 0.018, Fig. 3b). The causes of medication errors are shown in Table 5. The most frequent cause was dose omission by healthcare professionals in both groups. No medication error had serious consequences.

**Table 5:** Causes of medication error related to pre-admission medication which occurred within 5 days after admission

Causes	Pre-initiation of intervention	Post-initiation of intervention
Healthcare professionals	18	11
Dose omission	9	4
Overdose	6	2
Administration of the wrong drug	2	2
Loss of drugs	1	2
Administration to the wrong patient	0	1
Patients	1	3
Total	19	14

### Multivariate logistic regression analysis performed to estimate factors associated with incidents related to pre-admission medication

Multivariate analysis indicated that pharmacist-led MR prior to admission was a significant protective factor against incidents related to pre-admission medication (odds ratio (OR), 0.3810; 95% confidence interval (CI); 0.156–0.9320, *p* = 0.035) (Table 6). There were no significant differences in prescriptions for medications taken prior to admission by patient age.

**Table 6:** Factors associated with incidents related to pre-admission medication

Factor	Odds ratio	95% confidence interval	*p*
Pharmacist-led MR prior to admission	0.381	0.156–0.932	0.035
Prescription for medications taken prior to admission	0.784	0.226–2.730	0.703
Patient age	1.200	0.563–2.540	0.642

## Discussion

In this study, we showed that the incidence of medication errors related to pre-admission medication which occurred during hospitalization was reduced after the initiation of pharmacist-led MR for pre-admission patients. In the process of pre-admission MR, healthcare professionals collect BPMH with the involvement of patients, carers, primary care physician and/or pharmacies, and adapt it to prescriptions during hospitalization. Medication errors often occur at transitions of care due to discrepancies in medication information [5, 12–14]. In this regard, several studies have evaluated pharmacists’ clinical interventions in hospitalized patients [15–17]. We previously reported the benefits of pharmacist intervention on adverse events in hospitalized patients in the otolaryngology ward and in the respiratory medicine ward [18, 19]. Here, we evaluated the impact of pharmacist-led MR for pre-admission patients on the incidence of medication errors in the early post-admission period.

In this study, 94.2% of inpatients in the post-initiation group received pharmacist-led MR prior to admission. The remaining patients did not have the opportunity to visit an outpatient clinic before admission due to emergency admission or transfer from another hospital, and received MR after admission. After initiation of the intervention, physicians were able to increase the number of prescriptions which were alternatives to the pre-admission medication and which were required during hospitalization the day before admission, based on the medication history prepared by the pharmacist. As a result, the efficiency of post-hospitalization procedures, such as nurse receipt of physicians’ orders and preparation of medicines in the pharmacy department, was considered to have improved. The incidence of medication errors decreased significantly after the initiation of pharmacist-led MR for pre-admission patients, despite an increase in the number of inpatients. In particular, the number of errors related to administration by healthcare professionals decreased. Pharmacist-led MR prior to admission was a significant protective factor against incidents related to pre-admission medication. In the present study, it was not necessarily the case that pharmacist-led MR directly influenced prevention of medication errors or change of prescriptions. However, medication errors analyzed in this study were related to medications prescribed pre-admission only, and occurred in the early phase of hospitalization. It was considered that pharmacist-led MR for pre-admission patients indirectly contributed to a reduction in medication errors via the acceleration of tasks related to medicines during hospitalization.

The primary outcome of this study was the number of medication errors related to pre-admission medications that occurred within five days of admission. In an exploratory case study of 30 patients, Frydenberg et al. reported that the majority of medication errors occurred on admission, and that half of these were due to an incomplete medication list on the referral letter for hospitalization [20]. In a retrospective observational study, Dei Tos et al. reported the identification of unintentional medication discrepancies on admission in 53 of 144 patients [21]. Accordingly, transitions in care – such as admission – are associated with a risk of medication error and adverse events. Several reports have investigated the effect of MR performed early in hospitalization. In a study of older patients, Mazhar et al. reported that the most significant predictors of unintentional medication error were the number of medications prescribed on admission (OR 1.32, 95% CI 1.09–1.54, *p* < 0.034), number of sources consulted to obtain a better medication history (OR 1.53, 95% CI 1.38–1.76, *p* < 0.01), and completion of a medication history within 24 h of admission (OR 0.89, 95% CI 0.86–0.94, *p* < 0.03) [21].

Ouweini et al. reported the impact of pharmacist-led MR within 48 h of orthopedic admission for surgical treatment [22], and found that 84.5% of interventions based on pharmacist-led MR were accepted by surgical residents and fellows. In contrast, in a prospective cohort study of adult patients admitted to the emergency department, pharmacist-led MR was conducted prior to the preparation of a physician’s admission order. The findings indicated that serious errors occurred at similar proportions in the intervention and control groups [23]. Trends in clinical benefit were inconsistent across these reports. Possible reasons for this include differences in departments, age groups and study design. In a meta-analysis study evaluating the impact of MR at transitions of care, Redmond et al. reported that the implemented interventions reduced the number of medication discrepancies at transitions of care. However, they also noted that the quality of the evidence was very low [24]. Studies about MR have reported the effects of different interventions, practice processes and practice systems. Our present study is the first to evaluate the impact of pharmacist-led MR on pre-admission patients. Pharmacist-led MR in pre-admission patients contributed to a high proportion of acceptance by physicians of recommendations regarding antithrombotic medicine interruption and patient safety after admission.

This study has several limitations. First, it was conducted under a retrospective design. Factors that may affect work efficiency and patient safety after admission, such as differences in staffing structure before and after the initiation of the intervention, were not fully considered. There were no changes in the fixed number of physicians, nurses, or pharmacists on the orthopedic ward during the study period. In particular, the number of pharmacists assigned to the ward remained constant at two throughout the period. However, it was difficult to account for detailed transfers of physicians and nurses during the study period. Second, changes in the time taken to obtain medication histories were not reflected in the assessment of post-admission work efficiency or patient safety. Third, potential medication errors were not detected because the medication errors analysis was based on spontaneous reports from healthcare professionals. In general, the proportion of medication errors reported in previous reports varies depending on how the errors are detected [13].

## Conclusion

In this study, we found that pharmacist-led MR prior to admission contributed to a reduction in medication errors in the early post-admission period. Accurate MR by pharmacists early after admission is important in reducing medication discrepancy errors and improving patient safety.

## Data Availability

Not applicable.
